# First report of Lyme disease in Nepal

**DOI:** 10.1099/jmmcr.0.005128

**Published:** 2018-01-10

**Authors:** Sher Bahadur Pun, Sumit Agrawal, Santoshananda Jha, Lila Nath Bhandari, Bimal Sharma Chalise, Abadhesh Mishra, Rajesh Shah

**Affiliations:** Sukraraj Tropical and Infectious Disease Hospital, Teku, Kathmandu, Nepal

**Keywords:** Lyme disease, western blot test, doxycycline, Nepal

## Abstract

**Introduction:**

Lyme disease is a tick-borne illness caused by the spirochete *Borrelia burgdorferi* and is widely reported in the USA, Central Europe, South East Asia and Latin America. Until recently, no scientific report regarding Lyme disease in Nepal had been published.

**Case presentation:**

A 32-year-old, previously healthy female visited the hospital with a history of joint pains, fatigue, neck stiffness, tingling sensation and headache. She was initially treated for typhoid fever, brucellosis and malaria, but did not show significant improvement. Doxycycline was prescribed empirically for 3 weeks for the treatment of suspected tick-borne illness. A two-tiered immunoglobulin laboratory testing confirmed *Borrelia burgdorferi*. She developed post-treatment Lyme disease syndrome after completion of antibiotic therapy.

**Conclusion:**

To the best of our knowledge, this is the first report of Lyme disease in Nepal and probably the first documented case of post-treatment Lyme disease syndrome in Asia. Lyme disease might have been overlooked in Nepal and, therefore, patients having clinical signs and symptoms similar to Lyme disease should not be disregarded in differential diagnosis.

## Introduction

Lyme disease is one of the most known tick-borne infections worldwide [1]. It was first identified and reported in 1977 in the USA, when an epidemic form of arthritis occurred in the town of Old Lyme, eastern Connecticut [2]. Since then, it has been commonly reported in the USA, Europe, South East Asia and Latin America.

Lyme disease is caused by the spirochete *Borrelia burgdorferi* and is transmitted by the bite of an infected *Ixodes* tick [1]. In its early stage, it shows flu-like symptoms, and later spreads to joints, the cardiovascular system and nervous system. Erythema migrans (EM) is a unique hallmark of Lyme disease. Nearly one-third of patients with Lyme disease develop post-treatment Lyme disease syndrome (PTLDS) even after antibiotic therapy, which is often characterized by persistent or recurrent fatigue, joint pains and cognitive dysfunction [3]. The majority of people infected with *Borrelia burgdorferi*, however, cannot remember being bitten by a tick or suffering erythema migrans [4], and clinical symptoms induced by *Borrelia burgdorferi* are similar to those of other infectious diseases. Thus, the clinical diagnosis is followed by a two-tiered serological testing to confirm the disease [5]. There are few published reports describing Lyme disease in Asia [6, 7], while no cases of PTLDS have yet been described. We herein present the first laboratory-confirmed report of Lyme disease in Nepal.

## Case report

A 32-year-old female, resident of Pokhara city, district of Kaski, presented to our out-patient department in June 2017 with a history of joint pains, fatigue, neck stiffness, tingling sensation and headache. Earlier, in December 2016, she presented to a local hospital with high-grade fever associated with chills and rigors, joint pains, headache and bodyache. The laboratory tests revealed a total leuckocyte count of 9 800 mm^−3^, with neutrophils 77 % and lymphocytes 20 %. Her platelet count was 250 000 mm^−3^. Test results for liver and renal functions were found to be within normal limits. Urinary testing showed no evidence of bacterial infection. The Widal test was found to be positive. Based on a positive Widal test report, treatment was started with injectable ceftriaxone. The patient, however, did not benefit from antibiotic therapy. She tested negative for malaria. Brucellosis was suspected based on a positive brucella antibody test result, although a PCR test was negative. Doxycycline-rifampicin combination therapy was administered. Despite treatment, although fever subsided, she continued to have severe fatigue, joint pains, neck pain, tingling sensation and headache. She, therefore, decided to visit higher centres in India for further evaluation. She was then suspected of having tick-borne illness and, hence, was tested for *Borrelia burgdorferi* (Lyme disease), *Bartonella* (Bartonellosis) ehrlichiosis and *Babesia* (Babesiosis) using ELISA, Western blot (WB) and PCR (Table 1). She was treated empirically with a 3-week course of doxycycline. Besides *Borrelia burgdorferi*, all other tests were found to be negative. ELISA, WB and PCR confirmed *Borrelia burgdorferi*. ELISA for *Borrelia burgdorferi* showed a high level of IgM (22.9 U ml^−1^; ref. range: negative <12, positive >20) and an equivocal level of IgG (14.9 U ml^−1^; ref. range: negative <12, positive >20), respectively. On the IgM WB, the patient was positive for bands 23–25(+), 30(+), 31(+), 34(+), 41(++) and 83–93(+). On the IgG WB, she was positive for bands 18(+), 31(indeterminate), 34(+) and 41(+). The patient met both IGeneX and CDC/NYS WB criteria for IgM. The IGeneX criterion for IgG was positive but not enough to meet the CDC/NYS IgG criterion. A positive PCR result confirmed *Borrelia burgdorferi*. Electrocardiography (ECG), echocardiogram (EHO), magnetic resonance imaging (MRI) of the head, computed tomography (CT) of the chest, and abdomen and pelvis scan were unremarkable. The patient recalled a ring-like rash on her left thigh but did not remember a tick bite. Based on the clinical history (including the ring-like rash) and laboratory results, she was diagnosed with post-treatment Lyme disease syndrome.

**Table 1.** Diagnostic tests and results

***Borrelia burgdorferi***			***Bartonella* (FISH)**	**Ehrlichiosis**	***Babesia*** **(FISH)**
ELISA	IgM	IgG			
	Positive	Equivocal	Negative	Negative	Negative
WB					
IGeneX	Positive	Positive			
CDC/NYS	Positive	Negative			
PCR	Positive				

FISH, fluorescence in-situ hybridization.

## Discussion

Lyme disease is caused by the spirochete *Borrelia burgdorferi* and is transmitted to humans by the bite of *Ixodes* ticks. It produces flu-like symptoms and a distinctive circular bullseye-shaped rash that usually appears within 3–30 days after the bite [1, 8]. The presence of persistant symptoms after more than 6 months despite appropriate treatment is known as PTLDS [9]. In this condition, the majority of patients present with unexplained symptoms such as fatigue syndrome and joint or muscle pains. Therefore, it is possible that many Lyme patients are being missed or wrongly diagnosed in Nepal, as seen in this case. However, these patients are ultimately found to have rheumatologic, neurological or cardiovascular diagnoses, and hence it can be assumed that patients with Lyme disease are visiting specialty hospitals with late-stage presentations. In this report, our patient presented with fatigue syndrome, neurological problems and rheumatoid arthritis 6 months after completion of the treatment course. At this stage, the use of antibiotic therapy has not been proven to have beneficial effects, and is, therefore, not recommended for patients with PTLDS [10]. In this report, we did not prescribe any antibiotic for our patient. However, a two- to four-week regimen of doxycycline is the drug of choice for Lyme disease [10], while amoxicillin, cefuroxime axetil and ceftriaxone are other recommended antibiotics that can be used based on the stage of disease and infected organs [10].

*Borrelia burgdorferi* is the most commonly reported vector-borne disease in the USA. An estimated 300 000 cases of Lyme disease are reported in the USA each year [11]. In Europe, it is more frequently reported in central Europe [12]. However, only a few studies have previously documented PTLDS [3, 13], while no cases of PTLDS have yet been described in the Asia. Although *Borrelia burgdorferi* is one of the most commonly reported vector-borne diseases in the USA and Europe, there have been very few reports in Asia [6, 7]. In India, only a handful of Lyme disease cases have been published [6]. EM is estimated to occur in 70 to 80 % of infected patients and is used to suspect Lyme disease [8]. However, only approximately 25 % of the cases with Lyme disease had EM in Indian patients [6], while other clinical presentations are often non-specific with multisystem involvement. The absence of EM in south Asian patients could be a possible explanation of the marked differences in the incidence of Lyme disease between temperate and tropical regions. Nevertheless, comprehensive study is warranted to corroborate or refute this hypothesis.

Currently, a two-step laboratory test consisting of ELISA followed by WB test is required to confirm *Borrelia burgdorferi* [8]. However, these tests are not readily available in rural as well as urban areas of the least developed countries such as Nepal, meaning the actual number of Lyme disease cases may never be reported from these countries. As a consequence, they will receive far less attention from policymakers. Moreover, the WB test is expensive and requires a skilled person. The differences between IGeneX and CDC criteria often lead to considerable diagnostic confusion among clinicians. Thus, alternative diagnostic tools, uniformity of criteria and/or improved case definition may need to be developed, especially for resource-poor countries.

In conclusion, this is the first reported case of Lyme disease in Nepal to our knowledge, and probably the first documented case of PTLDS in Asia. Patients having clinical signs and symptoms similar to Lyme disease should not be disregarded in differential diagnosis. More research is needed to establish clinical and laboratory case definitions for resource-poor countries in order to allow early diagnosis and treatment to prevent complications from Lyme disease.
